# Systemic Immune-Inflammation Index (SII) of Patients With and Without Diabetic Neuropathy: A Cross-Sectional Study

**DOI:** 10.7759/cureus.87759

**Published:** 2025-07-12

**Authors:** Shubhransu Patro, Vibha Sharma, Arushi Choudhary, Yallambhotla Varuneil, Parmarth Arora, Sailendra Nayak, Jyoti Prakash Sahoo

**Affiliations:** 1 General Medicine, Kalinga Institute of Medical Sciences, Bhubaneswar, IND; 2 Pharmacology, Kalinga Institute of Medical Sciences, Bhubaneswar, IND

**Keywords:** complete blood counts, correlation analysis, diabetes type 2, diabetic peripheral neuropathy (dpn), impaired glycemic control, lymphocyte count, neutrophil count, npneutrophil-lymphocyte ratio (nlr), systemic immune-inflammation index (sii), total platelet count

## Abstract

Background and objectives: Diabetic neuropathy is a common microvascular complication of type 2 diabetes mellitus (T2DM), like diabetic retinopathy and nephropathy. The neutrophil count is raised during infection and inflammation, and the lymphocyte count is decreased in immunocompromised patients. The platelet count suggests the thrombotic and inflammatory conditions. Hence, the neutrophil-lymphocyte ratio (NLR) is the ratio of absolute counts of neutrophils to lymphocytes. The systemic immune-inflammation index (SII) is the product of NLR and platelet count. These indicate the immuno-inflammatory status of the patient. There is a dearth of literature assessing the SII of individuals with diabetic neuropathy. Hence, we mapped this study to evaluate and compare complete blood count (CBC), NLR, and SII of diabetic patients with and without neuropathy. We also correlated NLR and SII values of the study population.

Methods: This cross-sectional study was conducted from April 2024 to March 2025 at Kalinga Institute of Medical Sciences (KIMS), Bhubaneswar, India. We included adult diabetic patients admitted to the KIMS medicine ward. Hemoglobin, total leucocyte count (TLC), neutrophil count, lymphocyte count, platelet count, length of T2DM, and glycated hemoglobin (HbA_1c_) were all measured shortly after their hospitalization. NLR was calculated by dividing the absolute neutrophil count by the absolute lymphocyte count. We obtained the SII value by multiplying the platelet count by NLR. R software version 4.4.3 (R Core Team, Vienna, Austria) was used for statistical analysis.

Results: One hundred ninety-three diabetic patients were found eligible for this study. Their median age was 66 (55-73) years. Of them, 92 (47.7%) were women. The study population's median white blood cell (WBC) was 9.88 (8.72-11.24) × 10^9^/L. The median counts of lymphocytes and neutrophils were 2214 (1720-2647)/µL and 6864 (5796-8256)/µL, respectively. The study population's median platelet counts were 250 (189-293) × 10^9^/L. The study population's median HbA_1c_ was 8.61 (7.57-9.31)%. The study population's median duration of diabetes mellitus was 11 (6-18) years. The study population's median SII and NLR were 755.3 (521.4-1002.2) × 10^9^ cells/L and 2.93 (2.40-4.21), respectively. NLR and SII values were positively correlated (r = 0.92, p < 0.001). Regardless of neuropathy, the association between NLR and SII was identical among the study subjects.

Conclusion: Diabetes patients without neuropathy and those with neuropathy had comparable CBC, NLR, and SII values. The results of our study cannot be extrapolated due to the limited sample size and failure to account for several variables, including concurrent medications, comorbidities, and length of hospital stay.

## Introduction

Neuropathy is the most common microvascular complication of type 2 diabetes mellitus (T2DM). Distal symmetric polyneuropathy, also known as diabetic neuropathy, is the most prevalent neuropathy seen in diabetics [1]. Diabetic neuropathy is a progressive loss of sensory function of the lower extremities. Approximately 50% of diabetic individuals experience this complication. A sustained euglycemic state delays the development of diabetic neuropathy [1-3]. Chronic hyperglycemia, advanced glycation end products (AGEs), insulin resistance, immune-mediated pathways, and inflammation contribute to the development of diabetic neuropathy [4,5]. All these factors lead to nerve ischemia, which is manifested as diminished sensitivity to temperature, pressure, pain, and vibration [5].

Tumor necrosis factor-alpha (TNF-α) and growth factors are nonspecific markers of neuropathy [6]. Biomarkers for diabetic neuropathy are categorized as follows: oxidative biomarkers such as adiponectin, enzymatic biomarkers such as ceruloplasmin, nicotinamide adenine dinucleotide phosphate (NADPH), and peroxisome proliferator-activated receptor-alpha (PPAR-α), and inflammatory biomarkers such as vascular endothelial growth factor (VEGF), monocyte chemoattractant protein-1 (MCP-1), and nuclear factor kappa-light-chain-enhancer of activated B cells (NF-kB) [7]. The cost and technological challenges of measuring these inflammatory biomarkers prevent their use in routine clinical practice [8].

White blood cell (WBC) counts, both total and differential, are sensitive measures of inflammation. The neutrophil-lymphocyte ratio (NLR) has become a new alternative measure to evaluate chronic inflammation [9,10]. A recent meta-analysis found that NLR is substantially elevated in individuals with diabetic neuropathy as compared to those with diabetes without neuropathy [9]. The pathophysiology of diabetic neuropathy has recently been attributed to the relationship between inflammation and immune system-related cells, such as neutrophils, lymphocytes, and NLR [10-12]. The systemic immune-inflammation index (SII) has been developed to predict the body's inflammation and immune balance more accurately than NLR. It is calculated as follows: (platelet count × neutrophil count)/lymphocyte count [13,14].

NLR requires neutrophil and lymphocyte counts, whereas SII additionally examines platelet levels. Therefore, we designed this study to assess and compare diabetic patients with and without neuropathy regarding complete blood count (CBC), NLR, and SII. We additionally correlated the study population's NLR and SII values.

## Materials and methods

Study design

This cross-sectional study was conducted from April 2024 to March 2025 at the Kalinga Institute of Medical Sciences (KIMS) in Bhubaneswar, India. Before beginning the study, we received ethical approval from the Institutional Ethics Committee (KIIT/KIMS/IEC/1752/2024, dated 25.03.2024).

Study criteria

We included adult patients (of either gender) diagnosed with T2DM per the American Diabetes Association (ADA) criteria [15]. We excluded patients with type 1 diabetes mellitus, active infection or inflammation, or malignancy. Pregnant women and those on drugs affecting platelet levels were also excluded.

Study procedure

We noted the patient data from their case sheets during their discharge from the hospital. The sociodemographics, i.e., age, gender, marital status, and socioeconomic status of eligible participants, were noted. The Kuppuswamy classification was employed to categorize the socioeconomic class of the participants [16]. We recorded the following parameters assessed just after their hospital admission: hemoglobin, total leucocyte count (TLC), neutrophil and lymphocyte counts, platelet count, glycated hemoglobin (HbA_1c_), and duration of diabetes. The principal investigator assessed the neuropathy of the recruited patients. We calculated the NLR and SII from the data collected. The normal range of NLR is 0.78-3.53 [17]. The normal range for SII is 161-701 × 10^9^ cells/L [18]. For ease of assessment, we grouped the participants based on the presence of diabetic neuropathy at the time of admission.

Statistical analysis

We used convenience sampling for this cross-sectional study. The data distribution's normality was gauged using the Shapiro-Wilk test. The summary statistics for the continuous data were the median and interquartile range (IQR). The categorical data was expressed as frequency and proportion. The continuous and categorical data were assessed using the Wilcoxon and chi-square tests, respectively. We used Pearson's correlation to evaluate the association between NLR and SII of the study population. We expressed the coefficients of correlations with a 95% confidence interval (CI). R software version 4.4.3 (R Core Team, Vienna, Austria) was utilized for data computation [19]. Statistical significance was set at a p-value ≤ 0.05.

## Results

During the study period, 1174 diabetic patients were admitted to the medicine ward. Six hundred ninety-one (58.9%) subjects had incomplete details in their case sheets. Two hundred ninety (24.7%) patients were denied permission to participate in the study. The remaining 193 (16.4%) individuals were recruited into this study. Table 1 illustrates the sociodemographic and clinical parameters of those 193 study participants. The study population had a median age of 66 (55-73) years. Of them, one hundred one (52.3%) participants were men. The majority of participants were married and belonged to the lower socioeconomic class. The median hemoglobin was 10.8 (9.8-11.9) g/dL. The median WBC of the study population was 9.88 (8.72-11.24) × 10^9^/L. The median neutrophil and lymphocyte counts were 6864 (5796-8256)/µL and 2214 (1720-2647)/µL, respectively. The median platelet count of the study population was 250 (189-293) × 10^9^/L. The median HbA_1c_ value of the study population was 8.61 (7.57-9.31)%. The median duration of DM of the study population was 11 (6-18) years. The median NLR and SII of the study population were 2.93 (2.40-4.21) and 755.3 (521.4-1002.2) × 10^9^ cells/L, respectively.

**Table 1:** Demographic and clinical parameters of the study participants The categorical data was expressed as frequency and proportion. The continuous data was shown as median and IQR. The normal ranges of NLR and SII are 0.78-3.53 and 161-701 × 10^9^ cells/L, respectively. IQR: interquartile range, WBC: white blood cell count, HbA_1c_: glycated hemoglobin, NLR: neutrophil-lymphocyte ratio, SII: systemic immune-inflammation index, DM: diabetes mellitus

Parameters	Total (N = 193)	Patients with neuropathy (n = 94)	Patients without neuropathy (n = 99)	p-value
Age (years)	66 (55-73)	73 (68-78)	57 (49.5-63)	<0.001
Elderly (age > 60 years)	120 (62.2%)	83 (88.2%)	37 (37.4%)	<0.001
Gender				
Male	101 (52.3%)	50 (53.2%)	51 (51.5%)	0.163
Female	92 (47.7%)	44 (46.8%)	48 (48.5%)	
Marital status				
Married	176 (91.2%)	88 (93.6%)	88 (88.9%)	<0.001
Unmarried	11 (5.7%)	2 (2.1%)	9 (9.1%)	
Divorced/widowed	6 (3.1%)	4 (4.3%)	2 (2%)	
Socioeconomic status				
Low	133 (68.9%)	61 (64.9%)	72 (72.7%)	<0.001
Lower middle	42 (21.8%)	20 (21.3%)	22 (22.2%)	
Upper middle	18 (9.3%)	13 (13.8%)	5 (5.1%)	
Hemoglobin (g/dL)	10.8 (9.8-11.9)	10.7 (9.6-11.7)	10.9 (10.0-12.4)	0.762
WBC (10^9^/L)	9.88 (8.72-11.24)	10.12 (8.94-11.66)	9.81 (8.65-11.11)	<0.001
Neutrophil count (per microliter)	6864 (5796-8256)	6895 (5940-8573)	6825 (5606-7960)	<0.001
Lymphocyte count (per microliter)	2214 (1720-2647)	2338 (1798-2770)	2086 (1699-2559)	<0.001
Platelet count (10^9^/L)	250 (189-293)	257.5 (197.3-292.8)	232 (181-298)	<0.001
HbA_1c_ (%)	8.61 (7.57-9.31)	8.59 (7.53-9.32)	8.65 (7.62-9.31)	0.831
Duration of DM (years)	11 (6-18)	18 (14-25)	6 (4-10)	<0.001
NLR	2.93 (2.40-3.21)	2.91 (2.42-3.85)	2.94 (2.37-4.44)	<0.001
SII (10^9^ cells/L)	755.3 (521.4-1002.2)	736.3 (522.4-1018.3)	796.3 (514.2-990.1)	<0.001

**Figure 1:**
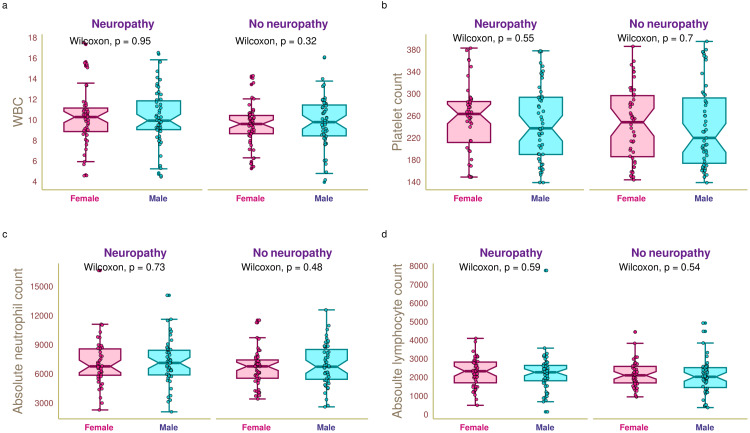
Complete blood count of the study participants The box-and-whisker and jitter plots illustrate complete blood counts of female and male participants with and without diabetic neuropathy. Panels a, b, c, and d demonstrate the white blood cell count (× 10^9^/L), platelet count (× 10^9^/L), absolute neutrophil count (/µL), and absolute lymphocyte count (/µL). The Wilcoxon test was employed to compare the intergroups (between female and male participants). WBC: white blood cell

Figure 1 illustrates the CBC of the study population. The median WBC of the study population was 9.88 (8.72-11.24) × 10^9^/L (Figure 1a). The median WBC values of female and male participants with neuropathy were 10.34 (8.92-11.21) × 10^9^/L and 9.99 (9.11-11.91) × 10^9^/L, respectively (p = 0.95). The median WBC values of female and male participants without neuropathy were 9.66 (8.72-10.49) × 10^9^/L and 9.84 (8.51-11.51) × 10^9^/L, respectively (p = 0.32). The median platelet count of the study population was 250 (189-293) × 10^9^/L (Figure 1b). The median platelet counts of female and male participants with neuropathy were 264.5 (212.8-287.0) × 10^9^/L and 238.5 (191.0-294.8) × 10^9^/L, respectively (p = 0.55). The median platelet counts of female and male participants without neuropathy were 249.5 (187.0-297.8) × 10^9^/L and 221 (175.0-293.5) × 10^9^/L, respectively (p = 0.70). The median neutrophil count of the study population was 6864 (5796-8256)/µL (Figure 1c). The median neutrophil counts of female and male participants with neuropathy were 6835 (5921-8631)/µL and 7189 (5952-8490)/µL, respectively (p = 0.73). The median neutrophil counts of female and male participants without neuropathy were 6834 (5611-7495)/µL and 6798 (5502-8574)/µL, respectively (p = 0.48). The median lymphocyte count of the study population was 2214 (1720-2647)/µL (Figure 1d). The median lymphocyte counts of female and male participants with neuropathy were 2361 (1736-2854)/µL and 2304 (1847-2673)/µL, respectively (p = 0.59). The median lymphocyte counts of female and male participants without neuropathy were 2140 (1730-2619)/µL and 2059 (1482-2554)/µL, respectively (p = 0.54). We did not find any statistically significant difference for any of these parameters.

**Figure 2:**
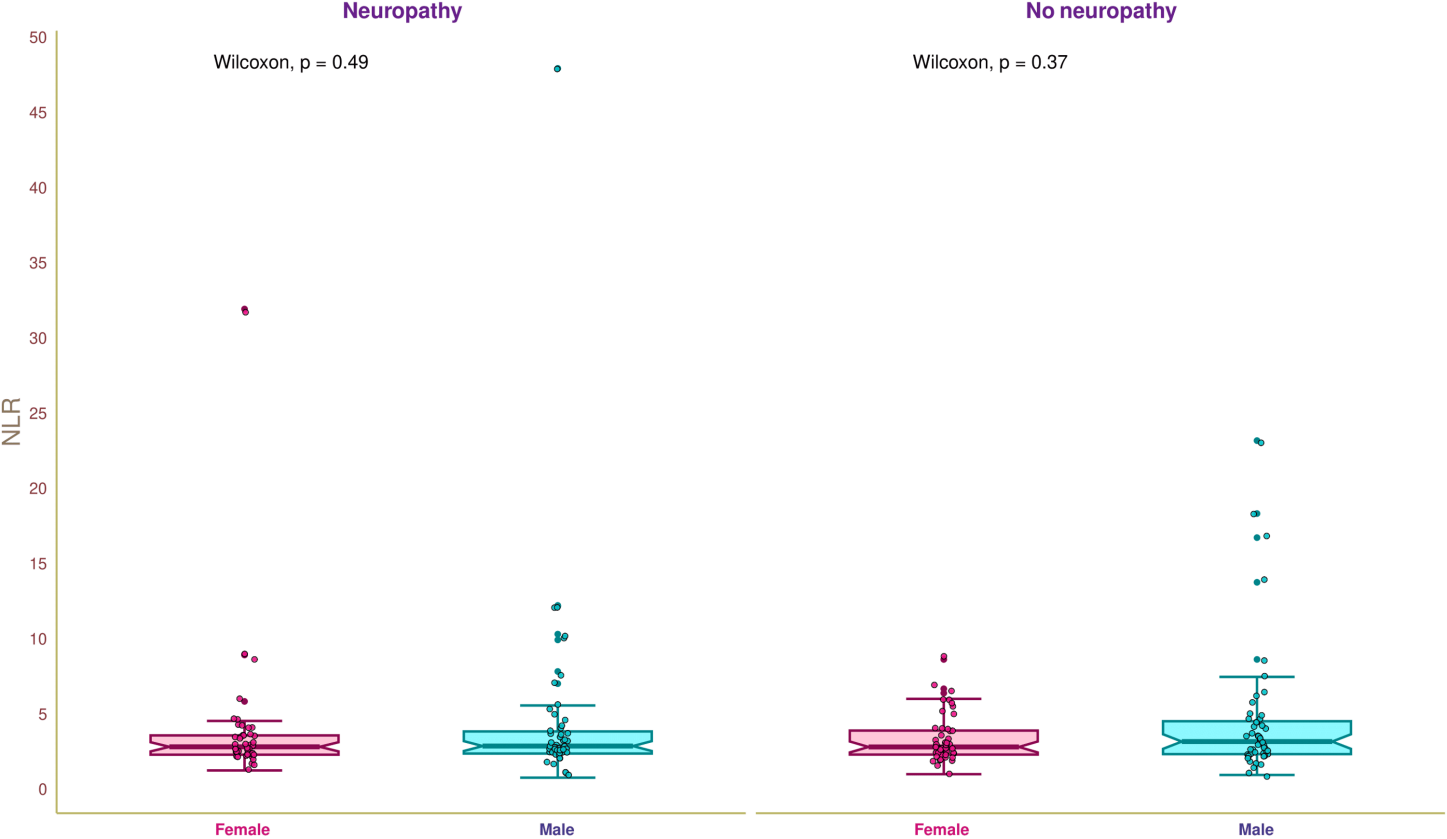
NLR of the study participants The box-and-whisker and jitter plots illustrate the NLR of female and male participants with and without diabetic neuropathy. The Wilcoxon test was employed to compare the intergroups (between female and male participants). The normal range of NLR is 0.78-3.53. NLR: neutrophil-lymphocyte ratio

Figure 2 illustrates the NLR of the study population. The median NLR of the study population was 2.93 (2.40-4.21). The median NLR values of female and male participants with neuropathy were 2.89 (2.37-3.65) and 2.94 (2.44-3.91), respectively (p = 0.49). The median NLR values of female and male participants without neuropathy were 2.88 (2.38-3.97) and 3.24 (2.41-4.60), respectively (p = 0.37). We did not find any statistically significant difference in the NLR of the study population.

**Figure 3:**
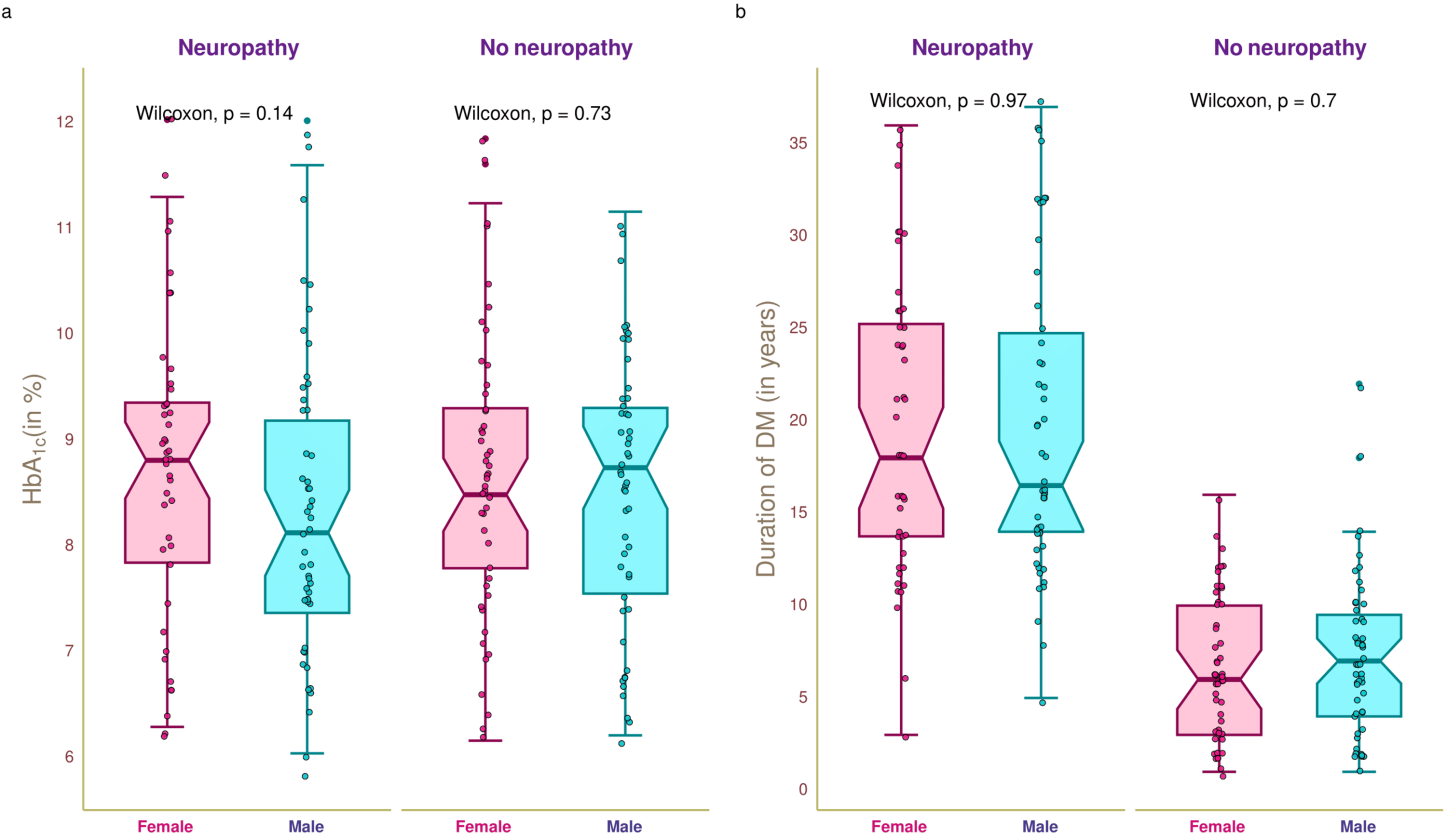
Glycemic status and diabetes duration of the study population The box-and-whisker and jitter plots illustrate glycemic status and duration of DM of female and male participants with and without diabetic neuropathy. Panels a and b demonstrate the HbA_1c_ value (in %) and duration of DM (in years). The Wilcoxon test was employed to compare the intergroups (between female and male participants). HbA_1c_: glycated hemoglobin, DM: diabetes mellitus

Figure 3 illustrates the glycemic status and diabetes duration of the study population. The median HbA_1c_ value of the study population was 8.61 (7.57-9.31)% (Figure 3a). The median HbA_1c_ values of female and male participants with neuropathy were 8.81 (7.84-9.36)% and 8.13 (7.37-9.19)%, respectively (p = 0.14). The median HbA_1c_ values of female and male participants without neuropathy were 8.49 (7.79-9.30)% and 8.74 (7.55-9.31)%, respectively (p = 0.73). The median duration of DM of the study population was 11 (6-18) years (Figure 3b). The median durations of DM of the female and male participants with neuropathy were 18 (13.8-25.3) years and 16.5 (14.0-24.8) years, respectively (p = 0.97). The median durations of DM of the female and male participants without neuropathy were 6 (3-10) years and 7 (4.0-9.5) years, respectively (p = 0.70). We did not find any statistically significant difference in the HbA_1c_ of the study population. However, the median durations of DM for the patients with and without neuropathy differed.

**Figure 4:**
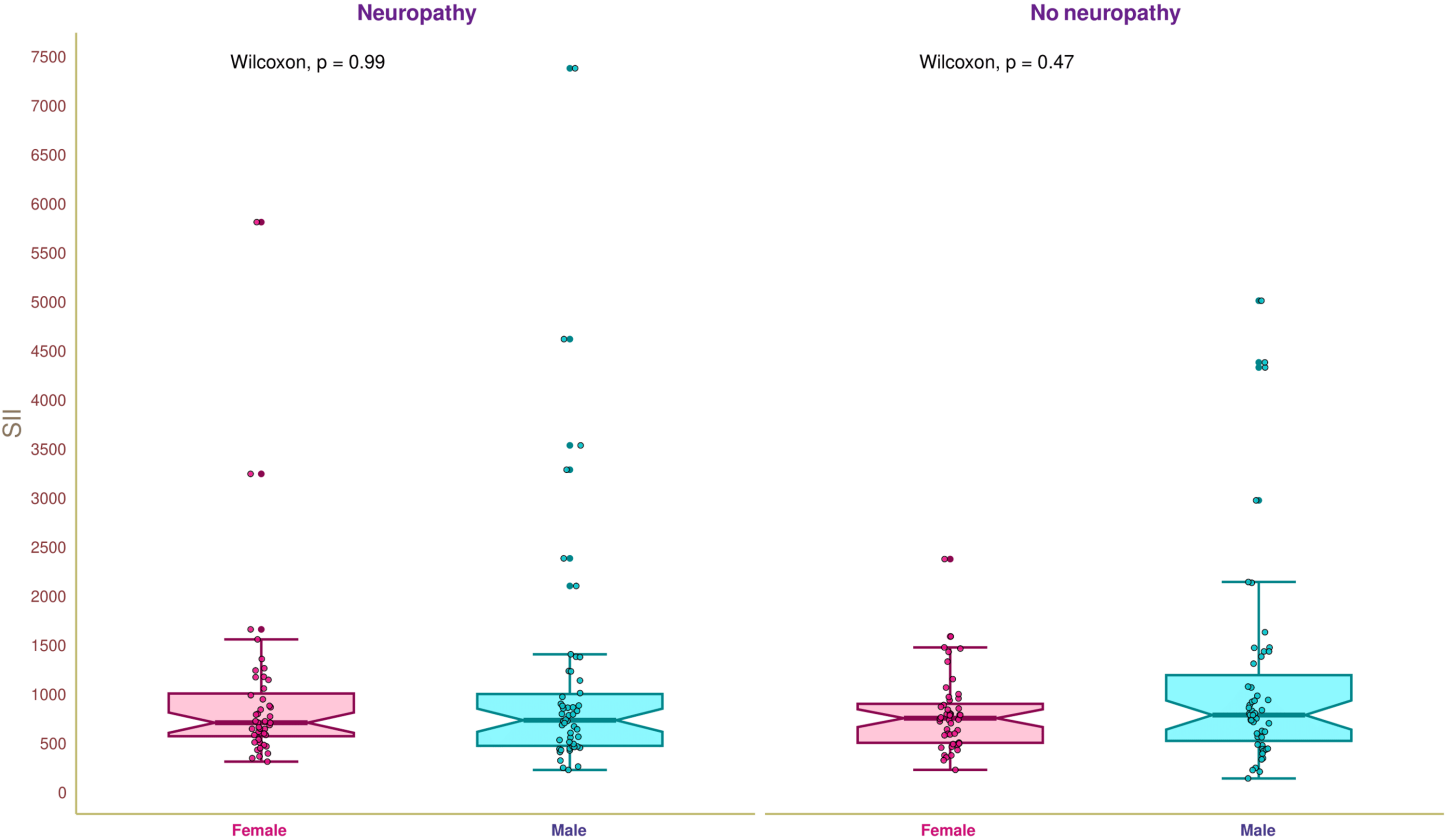
SII of the study population The box-and-whisker and jitter plots illustrate the SII of female and male participants with and without diabetic neuropathy. SII is calculated by multiplying platelet counts by NLR and is expressed as 10^9^ cells/L. The Wilcoxon test was employed to compare the intergroups (between female and male participants). The normal range of SII is 161-701 × 10^9^ cells/L. SII: systemic immune-inflammation index, NLR: neutrophil-lymphocyte ratio

Figure 4 illustrates the SII of the study population. The median SII of the study population was 755.3 (521.4-1002.2) × 10^9^ cells/L. The median SII values of female and male participants with neuropathy were 721.8 (584.0-1019.1) × 10^9^ cells/L and 746.8 (485.8-1014.1) × 10^9^ cells/L, respectively (p = 0.99). The median SII values of female and male participants without neuropathy were 767.6 (516.6-914.0) × 10^9^ cells/L and 799.7 (536.3-1206.6) × 10^9^ cells/L, respectively (p = 0.47). We did not find any statistically significant difference in SII values of the study population.

**Figure 5:**
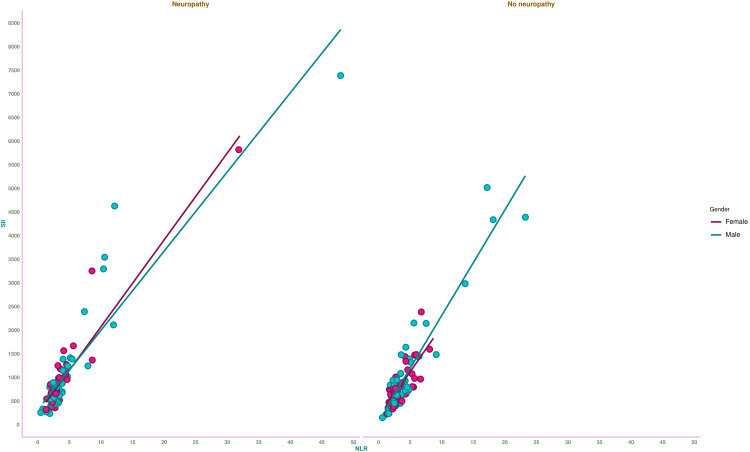
Correlation between NLR and SII of the study population The X- and Y-axes represent NLR and SII. Pearson's correlation test was used to check the association between NLR and SII values of female and male participants with and without diabetic neuropathy. NLR: neutrophil-lymphocyte ratio, SII: systemic immune-inflammation index

Figure 5 illustrates the correlation between NLR and SII of the study population. There was a strongly positive association (r = 0.92, 95% CI = 0.89-0.94, p < 0.001) between NLR and SII values. The association was similar among the participants regardless of the presence of neuropathy (those with neuropathy: r = 0.92, 95% CI = 0.88-0.95, p < 0.001, and those without neuropathy: r = 0.93, 95% CI = 0.89-0.95, p < 0.001). There were strong associations between NLR and SII values of female (r = 0.94, 95% CI = 0.90-0.97, p < 0.001) and male (r = 0.91, 95% CI = 0.85-0.95, p < 0.001) participants with neuropathy. There were also strong associations between NLR and SII values of female (r = 0.77, 95% CI = 0.63-0.87, p < 0.001) and male (r = 0.94, 95% CI = 0.90-0.97, p < 0.001) participants without neuropathy.

## Discussion

There are a few studies evaluating the SII and NLR values among patients with diabetic neuropathy. Hence, we conducted this cross-sectional study to determine and compare the SII and NLR values of patients with and without diabetic neuropathy. A total of 193 (16.4%) of 1174 diabetic patients admitted to the medicine ward were assessed. The study population had a median age of 66 (55-73) years. Of them, one hundred one (52.3%) participants were men. The median duration of DM of the study population was 11 (6-18) years. The median hemoglobin was 10.8 (9.8-11.9) g/dL. The median platelet count of the study population was 250 (189-293) × 10^9^/L. The median WBC of the study population was 9.88 (8.72-11.24) × 10^9^/L. The median neutrophil and lymphocyte counts were 6864 (5796-8256)/µL and 2214 (1720-2647)/µL, respectively. The median HbA_1c_ value of the study population was 8.61 (7.57-9.31)%. The median NLR and SII of the study population were 2.93 (2.40-4.21) and 755.3 (521.4-1002.2) × 10^9^ cells/L, respectively. Our findings concurred with those of the studies by Wan et al. [8] and Li et al. [20].

Neutrophils are the most abundant WBC in the blood, contributing to immune-mediated and inflammatory responses. Neutrophils increase in number in such conditions and tackle the tissue damage and inflammation by triggering cell lysis [21,22]. Lymphocytes elicit innate and adaptive immune responses to various inflammatory and infectious conditions [23]. Active platelets handle inflammation by engaging with leukocytes, inactivated platelets, and the endothelium [21,24]. WBC count and its constituents are easily measurable inflammatory markers [9]. NLR has a higher predictive value and is less impacted by neutrophil, lymphocyte, and total leukocyte counts [9,25]. A chronic inflammatory disease's two main constituents, i.e., high neutrophils and low lymphocytes, account for a higher NLR. High neutrophil and low lymphocyte counts suggest an ongoing inflammation and inadequate control of immunologic functions, respectively. Therefore, elevated NLR indicates the functioning of the immune system during long-term inflammation [9,25].

SII is a better indicator of immune-mediated and inflammatory conditions because it accounts for platelet count in addition to NLR [14,20]. We found no statistically significant difference between patients with and without neuropathy regarding the parameters assessed. Our findings were discordant with those of two recent studies [13,14]. A recent meta-analysis also found that diabetic patients with neuropathy had higher NLR values as compared to people with diabetes without neuropathy [9]. Insulin resistance in T2DM is linked to the activation of the immune system and inflammatory pathways, which can trigger microvascular complications such as diabetic neuropathy [26]. Recent studies have evaluated the SII and NLR values in individuals with diabetes [8,20,26-28]. All these studies advocated that SII values increase in diabetics as compared to nondiabetics. Our observations did not match those of the abovementioned studies [8,20,26-28], as there were no statistically significant differences regarding SII and NLR values of our study population.

The major strengths of our study were the analysis of NLR and SII values among diabetic patients with and without neuropathy. Our study has certain limitations as well. First, we could not further examine patients since their case files lacked sufficient information. Second, the effect of comorbidities and concurrent drugs on the patients' clinical characteristics and length of hospitalization was not evaluated. Third, we did not examine the follow-up studies or inquire further about them after they were discharged from the hospital.

## Conclusions

The CBC, NLR, and SII values of diabetic patients with and without neuropathy were similar. Except for the duration of DM, we could not find a statistically significant difference in any other parameters among the patients with and without diabetic neuropathy. Our study findings cannot be generalized owing to the small sample size and the lack of consideration of various factors such as comorbidities, duration of hospitalization, and concomitant drugs. We recommend prospective studies with a larger sample size to contrast NLR and SII in predicting the severity of diabetic neuropathy.
